# Role of Work and Family Factors in Predicting Career Satisfaction and Life Success

**DOI:** 10.3390/ijerph17145096

**Published:** 2020-07-15

**Authors:** Neena Gopalan, Murugan Pattusamy

**Affiliations:** 1 School of Business, University of Redlands, Redlands, CA 92373, USA; 2 School of Management, University of Hyderabad, Hyderabad 500046, India; pmmba@uohyd.ac.in

**Keywords:** work-family conflict, work role ambiguity, work-family balance, family satisfaction, job satisfaction, career satisfaction, life success

## Abstract

The mediating roles of work-family balance, job satisfaction and family satisfaction in work-family dynamics research has not been explored fully to delineate their probable intervening effects. Using spillover theory as the basis, the current study tests a model to identify the role of these factors in work-family conflict (and work-role ambiguity), career satisfaction and perception of life success. Responses obtained through an online survey from a final sample of 344 academic faculty, across different educational institutions in India, tend to suggest that work-family balance mediated work-family conflict and its potential influence on life success as well as career satisfaction, and also the relationship between work-role ambiguity and both life success and career satisfaction. While job satisfaction also showed similar results except for non-significant mediation between work-role ambiguity and life success, family satisfaction mediated only between work role ambiguity and life success. The importance of job satisfaction and work-family balance is highlighted in the context of reducing the negative impact of work-family conflict and work-role ambiguity on one’s career and life satisfaction. Results and their practical and theoretical implications, and future directions of research to further our understanding of work-family dynamics, etc., are discussed.

## 1. Introduction

Studies of the work-family interface issues started by focusing on the ‘conflict’ aspects between work and family domains [1]. In the past decade or so, research has been plentiful to endeavor also to comprehend the positive aspects emanating from work-family dynamics [2]. Investigators have studied a plethora of antecedents and outcomes related to work-family conflict and work-family balance/work family enrichment [3,4]. Studies have focused also on the role of work and family variables in predicting one’s job or family satisfaction or work-family balance [5]. However, what is evidently absent in the work-family dynamics literature are studies focusing on how work-related factors can possibly influence satisfaction in different domains of the academic faculty, such as career satisfaction or perception of success in one’s life. 

This paper focuses on the academic faculty who are exposed to a unique work-family dynamics environment compared employees doing a typical 8-5 job. Academic faculty often work well over 40 h/week. Teaching is habitually just one aspect of a university faculty job as the position also entails publication, service duties to the school, mentoring of students, etc. [6]. While studies of academic faculty emphasis stress and burnout [7], or factors that affect satisfaction such as income, role of superiors/leader characteristics, teaching self-efficacy, research expectations and pressure, etc. [8], a comprehensive project looking at how work and family factors may have a bearing on career satisfaction or perception of life success in the academic faculty is lacking. The current study intends to fill this gap using the tenets of Spillover theory as the foundation.

The purpose of this paper is to test the mediating role of family/job satisfaction as well as work-family balance in work-family conflict and work-role ambiguity and the outcome variables of career satisfaction and life success in the academic faculty. Below we list the reasons why this is recommended. 

First, the mediating role of family, job satisfaction or work-family balance on career satisfaction or on one’s perception of life success is sufficiently noteworthy to investigate and to comprehend how much they impact. To the best of our knowledge, such an inclusive analysis is lacking in the current literature. Second, what is the influence of work family conflict on career satisfaction and life success when gratification in one’s work or family, or a balance between the work and family domains, act as mediators? Again, current literature does not offer an answer to this question. Third, given the plethora of research focusing only on the potentially negative effect of work-role ambiguity, how does work role ambiguity play a role in predicting career satisfaction or perception of life success, mediated through job or family satisfaction or work-family balance? Fourth, an attentiveness to the above is important to have a more realistic and profound understanding of the interplay between work and family dynamics. The outcomes studied here are not confined to immediate job or family satisfaction or creating a balance between the two but also extends beyond to one’s professional satisfaction (career satisfaction) and perception of success in one’s life. Work and family conflict and work-role ambiguity may be prevalent in the lives of many professionals, including academic faculty. How can we reduce the negative consequences of these variables and/or bring about career satisfaction and a feeling of being successful in one’s life? We aim to find answers to such questions. Fifth, as this research is based in India, we trust this paper also sheds light on how a diverse cultural background may sway results that might be dissimilar from those found in Western studies. It is important that work-family studies branch out of the Western context and consider more local samples to comprehend the subtle variables more accurately and to be able to offer practical solutions to issues, keeping local dynamics in mind. Sixth, this explorative inquiry focuses on career/professional satisfaction as one of the outcome variables since the sample is an academic faculty who hold advanced degrees and for whom professional or career satisfaction is vital in addition to the more ‘proximal’ variable of job satisfaction. Finally, and possibly most significantly, work-family interface research must also contemplate additional variables, including profession-specific variables, to advance our comprehension of the real bearing of such variables and the nuances experienced by professionals. Having such clarity supports the crafting of policies to diminish the effect of work-interference-family or work ambiguity and instead provide the required equilibrium and positive spillover that might contribute to a happier and productive labor force.

## 2. Theoretical Framework and Hypotheses Development

### 2.1. Theoretical Framework

Primarily, we have based our hypotheses on the arguments of Spillover [9] and Person-Environment theories. As per the spillover model, experiences (including skills, attitudes, behaviors and even emotions) in one domain can affect one’s functioning including mood, perception and even behavior in the other domain, through a permeable boundary between the two domains. Spillover can produce positive or negative outcomes [10]. Thus, it is highly probable that feelings of work conflicting with the contradictory domain of family can result in less family satisfaction. Perceiving conflict can also produce a negative affect that can influence work-family balance, defined as a simultaneous perception of low conflict and high enrichment [11]. Such a definition combining both positive and negative experiences of work-family balance is based on a combined ‘spillover’ approach [5]. Thus, sensing conflict can automatically have an effect on ‘balance’ perception, albeit in an unhealthy and unproductive manner, reducing the positive emotions often associated with having a sense of balance. 

Person-Environment (P-E) theory proposes that the interaction between an individual and his/her environment affect consequences. One version of P-E fit theory, ‘supplies-values fit’, refers to the fit between personal motives/goals with supplies in a given environment [12]. ‘Supply’ refers to ‘resources/rewards’ in the environment and those emanating from the person’s experiences in the environment. This approach has been used to investigate the relationship between environment fit and consequences such as job satisfaction, happiness, etc. [13]. Work-family conflict can signal a lack of fit between one’s goals and the resources available in the environment, leading to lesser job satisfaction.

Similarly, poor work-role satisfaction also reduces work-family balance since, according to the definition above, ambiguity can lead to frustration, feelings of liability, and dearth of enthusiasm. Such feelings and attitudes in the broad work domain can impact low job satisfaction, as per the P-E fit theory. It can also spillover negatively to the family domain. Work-family balance involves both positive and negative dimensions. A rise in negative dimension, emanating from experiences of work-role ambiguity, can diminish the positive side, such that individuals report less work-family balance. 

Satisfaction in the family can spillover to one’s professional life influencing its satisfaction. Likewise, job satisfaction, by virtue of PE theory, can also play a role in defining career satisfaction as resources available in one’s job and one’s work experiences can progressively have a bearing on this. Finally, having a sense of balance between one’s work and family alludes to the positive side of work-family balance playing a role in inducing more career satisfaction and a higher perception of being successful in one’s life. 

We also refer to the *domain importance* approach and the *subjective well-being* concept to explain our hypotheses involving career satisfaction and perception of overall success in life. The domain importance approach emphasizes the relative standing an individual attributes to various life domains [14]. Individual distinctions exist in the prominence given to different domains in one’s life [15] which can have a bearing on one’s perception of life success or career satisfaction. The subjective well-being concept refers to how people evaluate specific domains in their lives [16]. This consists of judgments of satisfaction, as well as disagreeable occurrences in one’s life, which, in turn is affected by individual and contextual factors such as one’s work, family, etc. From these perspectives, one can argue that career satisfaction and overall sense of life success can be contingent on the significance one gives to career or overall life welfare and the evaluation of the level of both positive and negative experiences in one’s life. Thus, having less work-family conflict or work ambiguity, or a high level of satisfaction in one’s family or work, or a sense of balance between the two domains, may predispose the individual to evaluate their career and overall life success in a specific [positive] manner more than otherwise. 

Below, we provide background information on each of the variables and list our hypotheses.

### 2.2. Work-Family Conflict, Family Satisfaction, Job Satisfaction, Work-Family Balance, Career Satisfaction and Life Success

*Work-family conflict (WFC)* is defined as an inter-role conflict where the role pressures from work and family domains are incompatible [17]. Numerous studies depict how WFC can lead to negative consequences such as strain, low family satisfaction, burnout, etc. [18]. Technological advances may mean that work cannot be switched ‘off’ outside the place of employment, and that academic faculty often tend to report high work-family conflict [19]. This can spillover to their family lives or influence a realistic self-assessment of their job, or even their discernment of success in their lives.

*Family Satisfaction (FS)* is a contributing element to overall happiness in life [20]. Although studies suggest that rotating work shifts can disrupt family lives [21] or that work-family conflict can have a negative effect on one’s family satisfaction [18], few studies have considered the mediating role of family satisfaction in predicting outcomes, and even fewer its influence on career satisfaction or on perception of overall life success. The direct consequences of work-family interface variables have been the focus of most work-family studies with fewer studies attempting to understand the underlying mechanisms through meditating variables [22].

*Job Satisfaction (JS*). The literature on work-family at times implicitly assumes that work is constrained to a typical 8-5 schedule [23], which unfortunately is in contrast to circumstances in the lives of academic faculty. Work-family conflict can have a negative bearing on job satisfaction [24]. Some studies allude to the positive relationship between work-family balance and job satisfaction [25]. Nevertheless, an academic faculty position is associated more with a career/profession, particularly for university faculty who hold terminal degrees such as a Doctorate. Hence it is imperative also to study career satisfaction, but not many studies, including those of professionals such as academic faculty, have encompassed career satisfaction as a variable under consideration. 

*Work-family balance (WFB)*. The positive influence of work on family has been studied under diverse perspectives of work-family balance, work-family enrichment, etc. [5]. An inability to balance work and family domain duties runs the risk of being detrimental to even the most accomplished employee [24]. To our knowledge, few studies [26] have attempted to understand the mediating role of work-family balance. Studies are even scarcer on the bearing of work-family conflict on work-family balance. However, unlike the proposed study detailed below, Chan et al. (2016) focused on the role of work-family balance on two commonly studied outcomes: family and job satisfaction [22]. We maintain that work-family conflict can have a negative relationship not only with family or job satisfaction but also with academic faculty experiencing the much needed ‘balance’ between their work and family. Further, while work-family conflict can negatively relate to job satisfaction, family satisfaction and work-family balance, we posit that it also individually relates to outcomes such as career satisfaction and perception of life success. 

*Career satisfaction (CS*) is identified as satisfaction with advancement in one’s occupation, a salary hike, procurement of new skills, etc., and is an important factor in valuing a person’s career as a whole [27]. Thus, it comprises positive work and psychological outcomes emanating from work experiences. It has been researched in diverse contexts such as work-family balance in female professions [28], career mentoring [29], etc. Nonetheless, examinations of the mediating relationship of WFB or family or job satisfaction on CS have been deficient. The closest we found was a study of the direct effect of WFC on career satisfaction of dual earning couples [30] and of work-family enrichment on career satisfaction [31]. However, comprehensive assessment of the probable effect of WFC, or the mediating role of satisfaction or work family balance, on career satisfaction of faculty members is lacking. 

*Life Success (LS*) has been used as a term to refer to an individual’s perception of success in their lives [32]. This discernment can be evident through satisfaction in different areas of one’s life such as work, family, social circle, etc. Perception of life success can thus be influenced in the lives of academic faculty by different work and family related factors, or those that overlap in both domains. For example, as noted earlier, work-family conflict can have a bearing on personal life satisfaction or job satisfaction which, in turn, may very well affect how one views his/her success in life. Life success has been studied in research focusing on motivation, personality traits, etc. [33], but not adequately in studies involving work-family in the academic faculty.

*Mediating role of family satisfaction (FS), job satisfaction (JS) and work-family balance (WFB) between work-family conflict (WFC) and career satisfaction (CS) as well as, between work-family conflict (WFC) and perception of Life Success (LS)*

Individuals enduring conflict in allocating adequate time and energy (because of work) to their family are likely to experience a lower level of family satisfaction as WFC disrupts satisfactory engagement in one’s family responsibilities which can spur family dissatisfaction. WFC can negatively affect individual’s life stress according to Parasuraman et al. (1996) [34]. Such stress can unproductively affect JS [35] and WFB, as well as spur a low sense of happiness and contentment in one’s life [36].

Having a well-adjusted involvement in multiple roles can possibly produce positive outcomes in both work and family domain for individuals [37]. Studies have found that WFB positively influences fulfillment in the overall life of individuals and have reported on the positive role of JS in work and family domains [38,39] and on the constructive aspect of FS in work and overall life [40]. Employees tend to perceive their life more favorably and with a constructive frame of mind if WFC tends to be less [41].

We postulate that having a sense of equilibrium between work and family or a sense of fulfillment in one’s job or family is vital to reduce the possible negative impact of WFC in one’s life. Thus, finding ways to increase FS, JS and WFB can lessen the potential effect of WFC on CS. That is, though work and family may come into conflict, family satisfaction, job satisfaction or work-family balance, which could also be accounted for by other factors (such as having an understanding spouse or supportive colleagues, respectively) could reduce the undesirable results of such conflict and in turn lead the way to the employee viewing life from a positive frame of mind and also experiencing higher CS. The mediating role of WFB or FS have not been studied as extensively as they should be. We thus propose that a sense of satisfaction in one’s family or job or an overall sense of feeling of balance between the two can lead to less conflict between work and family responsibilities, affecting the overall sense of success in one’s life or CS. Such propositions need to be tested to further understand how much having FS or JS or WFB can mitigate the probable adverse bearing of WFC on one’s CS or the perception of one’s life as being successful.

Based on this evidence, we posit that WFB, FS and JS can mediate between WFC and CS, and also between WFC and LS. That is, though WFC leads to low FS, JS, WFB, and CS as well LS, we maintain that FS, JS and WFB can act as ‘independent’ variables to improve [experience a higher] CS and sense of life success. Our H1 and H2 follow these premises:

**Hypothesis** **1.** *Family satisfaction (H1a), job satisfaction (H1b) and work-family balance (H1c) mediate the relationship between work-family conflict and career satisfaction.*

**Hypothesis** **2.** *Family satisfaction (H2a), job satisfaction (H2b) and work-family balance (H2c) mediate the relationship between work-family conflict and perception of life success.*

### 2.3. Work-Role Ambiguity, Family Satisfaction, Job Satisfaction, Work-Family Balance, Career Satisfaction and Life Success

*Work-role ambiguity (WRA*) results when an employee is uncertain of the responsibilities and duties expected of one’s position [42]. Such vagueness can lead to stress and strain with far-reaching consequences for one’s work and family lives [43]. In the lives of academic faculty, WRA may be due to deficiency of adequate mentoring especially early in one’s career, or tenure pressure [44] mostly stemming from the need to be research prolific, etc., that can affect fulfilment not only in one’s job, but even in the family and possibly influencing how one is able to effectively balance work and family roles. It is probable that such damaging experiences may also have a distal influence on insight into how one’s career is progressing or on evaluation of success in life.

*Mediating role of family satisfaction (FS), job satisfaction (JS) and work-family balance (WFB) between work-role ambiguity (WRA) and career satisfaction (CS) as well as, between work-role ambiguity (WRA) and perception of Life Success (LS)*

Unpredictability associated with one’s work can not only impair one’s JS [45], but also can contribute to a source of stress relating to one’s family and work domains. Continued stress can also impair the sense of feeling successful in one’s life [46] and can be a source of work damaging satisfaction in family life [47] including strains in the family [48]. Role ambiguity can also lead to higher anxiety [49] which in turn can reduce a sense of achievement and well-being in one’s life [50]. 

Using arguments similar to H3, we maintain that a sense of JS and FS as well a feeling of balance between work and family domains can potentially mitigate negative consequences of WRA and instead improve one’s perception of CS and LS. It is imperative for academicians to experience more satisfaction in one’s job and family and have an improved feeling of balance between work and family, as this can reduce the damaging aspects of work role ambiguity on one’s CS or perception of LS. We propose that JS, FS and WFB can lessen the negative relationship between WRA and CS (and LS) as these three intermediators can lead to better CS and LS for reasons mentioned earlier. On the basis of these arguments we postulate the following two hypotheses.

**Hypothesis** **3.** *The relationship between work role ambiguity and career satisfaction is mediated by family satisfaction (H3a), job satisfaction (H3b) and work-family balance (H3c).*

**Hypothesis** **4.** *The relationship between work role ambiguity and perception of life success is mediated by family satisfaction (H4a), job satisfaction (H4b) and work-family balance (H4c).*

**Figure 1:**
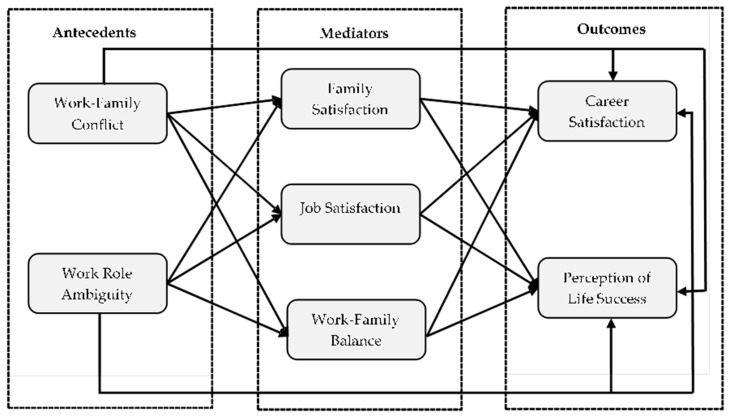
The conceptual model.

Figure 1 illustrates our conceptual model.

## 3. Method

### 3.1. Data Source and Description

A cross-sectional design and survey method were utilized to collect data from academicians working in Indian institutions. Participants’ email identities were collected through institution websites and conference advertisements. Similar approaches were successfully used in earlier studies in the Indian context [26]. Around 3000 emails were sent to faculty members working in state-owned (public) and private educational institutions in South India, requesting their voluntary participation in an academic enquiry into work and family dynamics. The data were collected as part of the co-researcher’s dissertation studies in India, and necessary approvals were given by the Doctoral committee ensuring the proper code of ethical conduct was followed. Details of this conduct are explained below.

Data were collected using a self-reported online questionnaire. Before completing the survey, participants read a statement informing them of the purpose of the project, their rights to participate in the survey only if they were fully willing without any coercion and to stop completing the survey at any time for any reason without any penalty. They were also assured that no identifying information would be collected and only the researchers would have access to the data, that only aggregate results would be reported and that under no circumstances would there be any risk of revealing their personal identity or individual response. The criteria to participate in this survey was that the participants had to be employed full-time with an educational institution. Such full-time work criteria are recommended in studies of work-family conflict for these participants are more likely face the challenges of managing work and family duties [51].

The final response rate was 365 responses, out of which 21 were removed due to a large number of missing values. The final sample consisted of 344 responses yielding a response rate of 12.16%. We had 26 items in our survey. We have followed the 1:10 ratio criteria, which suggests that 260 responses would be required for multivariate data analyses in our case (i.e., 26 ∗ 10). We had 344 responses which was sufficient for running multivariate data analysis. After cleaning the data for outliers, missing data, etc., data from 344 academic faculty were considered as suitable for further analyses. Of the 344 responses, 64.8% were male, 70.1% were married, and 75.6% reported as working in private educational institutions. The average age was approximately 35 years while the average years of work experience was reported at approximately 10–11 years (i.e., 129.17 months).

### 3.2. Measures

Likert scale was used to measure all constructs (except for ‘life success’) used in the survey to collect data, 1 = strongly disagree to 5 = strongly agree. 

*Work-family conflict* was measured using five items developed by Netemeyer, et al. (1996) [1]. A sample item is “The demands of my work interfere with my home and family life”. 

*Work role ambiguity* was measured using six items developed by Rizzo, et al. (1970) [52]. A sample item is “I know that I have divided my time properly”. 

*Family satisfaction* was measured using the three items developed by Edwards, et al. (1999) [9]. A sample item is “My family life is very enjoyable”. 

*Job satisfaction* was measured using the three-item scale developed by Cammann, et al. (1979) [53]. A sample item is “All in all I am satisfied with my job”. 

*Work-family balance* was measured with the five items scale used by Hill, (2001) [54]. A sample item is “When I take a vacation, I am able to separate myself from work and enjoy myself”. 

*Career satisfaction* was measured with the scale used by Martins, et al. (2002) [55]. A sample item is “In general, I am satisfied with my career status”.

*Life Success*: The following item, by Martinengo, et al. (2010) [32], was used to measure life success: “All in all, how successful do you feel in your personal life”. It was measured using a five point Likert scale ranging between 1 extremely unsuccessful to 5 extremely successful. 

*Demographic/Control variables*: Gender was coded as (0 Female, 1 Male), Marital Status as (0 Unmarried, 1 Married), and Type of Occupation as (0 State-owned/Government educational institution, 1 Private educational institution). Age and number of years of work experience of participants were also included as control variables. 

### 3.3. Data Analysis

SPSS v21 software (IBM, New York, NY, USA) was used to compute descriptive statistics, zero-order correlation, and Cronbach’s alpha values, while AMOS v21 software (IBM, New York, NY, USA) was used to test the measurement model validity through a confirmatory factor analysis. Single factor to six factor models were performed using the steps recommended in the recent literature (e.g., Bai, et al., 2016) [56]. This procedure is considered as one of the more robust methods to establish discriminant validity and convergent validity. Goodness of Fit Index (GFI), Comparative Fit Index (CFI) and Root Mean Square Error of Approximation (RMSEA) were used to assess the model fitness values. We used Akaike information criterion (AIC) [57] for model comparison purposes. Finally, we tested the proposed hypothesized relationships using PROCESS macro add in package for SPSS software [58].

## 4. Results

Table 1 presents the descriptive statistics, zero-order correlations and Cronbach’s alpha values. Reliability values of all measurements ranged between 0.72 and 0.86, indicating good to very high reliability. 

A confirmatory factor analysis (CFA) was conducted on the self-reported scales (i.e., WFC, WRA, FS, JS, WFB and CS) to examine discriminant validity. Life success (LS) was excluded in this process as it is a single item measure. As presented in Table 2 below, CFA results rendered support for a six factor model as a better fit with the data, compared with other models (*χ*^2^ = 303.17, *df* = 154, GFI = 0.91, CFI = 0.94, RMSEA = 0.053). Since the hypothesized measurement model fits well with the data, the discriminant validity of the proposed theoretical model is well established. The measurement model fitness values are within the cut off limit. All factor loadings were significant at 0.01 significance level with loadings ranging from 0.53 to 0.87. 

Common method bias was addressed through the following analyses recommended in the literature [59]. First, we performed Harman’s single factor test where the first factor did not explain more than 50% of the variance. Second, the results of a single-factor model to six-factor model comparison revealed that common method bias was not an issue. As mentioned earlier, the six factor model was indeed the model that had the best fit with the data. Table 1 below depicts the descriptive zero-order correlations and reliability statistics, while the six factor model along with the other models (that did not have the best fit) are reported in Table 2. We also performed the variance inflation factor (VIF) analysis using the linear regression procedure in SPSS software. We ran two analyses, one, with life success as a DV and the second with career satisfaction as a DV. No issue of multicollinearity was evident in both analyses.

### Hypotheses Testing

As recommended by researchers [60,61], path analytic procedure was used to test the hypotheses and percentile bootstrap analysis to test the indirect effects. Further, a PROCESS Macro was utilized to estimate the indirect effects using 5000 bootstrap sample. The mediation model coefficients are reported in Table 3 for the mediation relationship between work-family conflict and career satisfaction (as well as life success) through family satisfaction, job satisfaction and work-family balance. Gender, age, marital status, type of educational institution and years of work experience were used as control variables. While analyzing H1, H2, H3 and H4, none of the control variables were found to be significantly related to the outcomes. 

**Figure 2:**
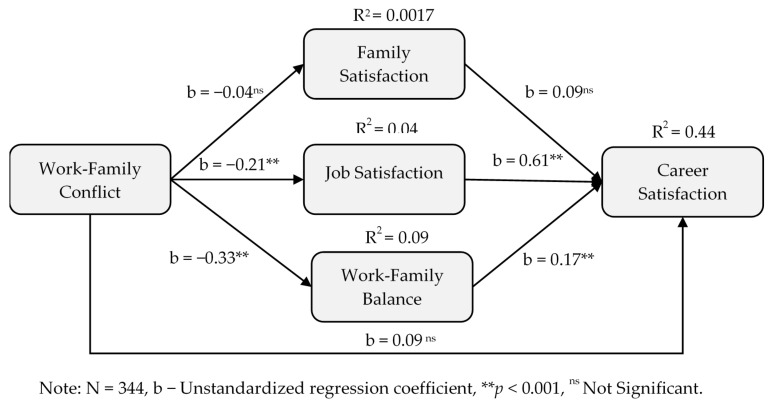
Mediational model on work-family conflict and career satisfaction.

H1 proposed that the relationship between work-family conflict and career satisfaction was mediated by family satisfaction (H1a), job satisfaction (H1b) and work-family balance (H1c). Table 3 illustrates results of mediational relationship between work-family conflict and career satisfaction (as well as between work-family conflict and life success). The results of the mediational relationship between work-family conflict and career satisfaction were significantly mediated through job satisfaction (H1b: indirect effect = −0.13, [−0.20, −0.07]) and work-family balance (H1c: indirect effect = −0.06, [−0.10, −0.02]), but not through family satisfaction (H1a: indirect effect = −0.004, [−0.02, 0.01]). Thus, H1b and H1c were supported, but not H1a, which suggests that only job satisfaction and work-family balance play a significant role in improving career satisfaction of faculty members via less work-family conflict. As is depicted in Figure 2 below, a direct effect between work-family conflict and career satisfaction was also not significant among H1b and H1c relationships, further indicating a full mediation effect. 

**Figure 3:**
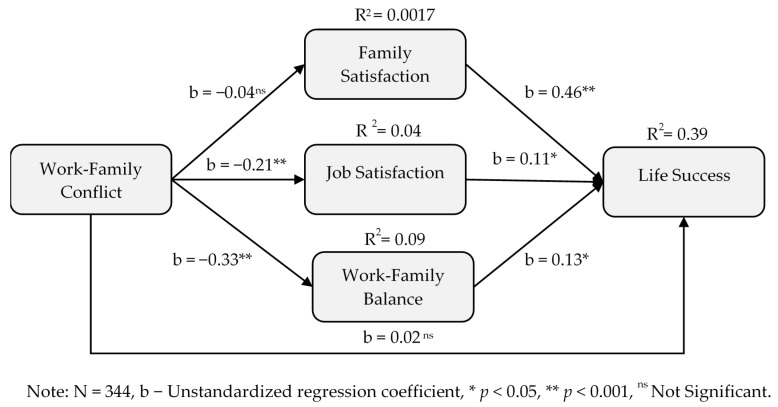
Mediational model on work-family conflict and life success.

Our H2 predicted that the relationship between work-family conflict and life success was mediated by family satisfaction (H2a), job satisfaction (H2b) and work-family balance (H2c). Table 3 above also includes the results of the mediational relationship between work-family conflict and life success, which was significantly mediated through job satisfaction (H2b: indirect effect = −0.03, (−0.06, −0.003)) and work-family balance (H2c: indirect effect = −0.04, (−0.08, −0.01)), but not through family satisfaction (H2a: indirect effect= −0.02, (−0.07, 0.03)). Thus, support exists for H2b and H2c, but not for H2a. These results suggest that job satisfaction and work-family balance can play significant roles in academic faculty experiencing improved life success through less work-family conflict, but family satisfaction does not seem to have a significant role in helping academic faculty experience improved life success by reducing work-family conflict. Further, as shown in Figure 3 below, direct effect work-family conflict on life success was non-significant, suggesting a full mediation effect only among the above mentioned significant mediation relationships. 

**Figure 4:**
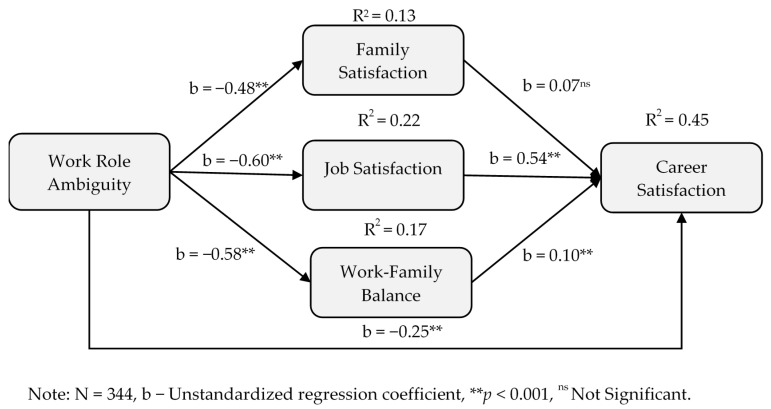
Mediational model of work role ambiguity and career satisfaction.

H3 proposed that the relationship between work role ambiguity and career satisfaction was mediated by family satisfaction (H3a), job satisfaction (H3b) and work-family balance (H3c). Table 4 below provides results of the mediational relationship between work role ambiguity and career satisfaction (and also between work role ambiguity and life success). As is shown, the mediational relationship between work role ambiguity and career satisfaction was significantly mediated through job satisfaction (H3b: indirect effect = −0.33, (−0.44, −0.23)) and work-family balance (H3c: indirect effect = −0.06, (−0.13, −0.001)), but not through family satisfaction (H3a: indirect effect = −0.03, (−0.09, 0.02)). This suggests support for H3b and H3c, but H3a is not supported. That is, H3 results tend to advocate that while JS and WFB are helping faculty to enhance their career satisfaction through reduction in WRA, FS does not play a significant role in improving their career satisfaction through less WRA. A partial mediation effect among these relationships was also confirmed through the significant direct effect (see Figure 4 below) for H3b and H3c. 

**Figure 5:**
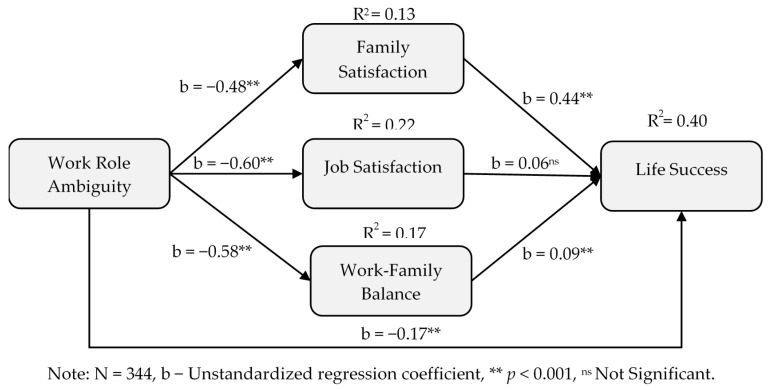
Mediational model on work role ambiguity and life success.

Hypothesis 4 proposed that the relationship between work role ambiguity and life success was mediated by family satisfaction (H4a), job satisfaction (H4b) and work-family balance (H4c). As presented in Table 4 above, the mediational relationship between work role ambiguity and life success was significantly mediated through family satisfaction (H4a: indirect effect= −0.21, [−0.29, −0.14]) and work-family balance (H4c: indirect effect = −0.05, [−0.11, −0.004]), but not through job satisfaction (H4b: indirect effect = −0.04, [−0.11, 0.02]). Therefore, we found support for H4a and H4c, but H4b is not supported. That is, the sense of life success of academic faculty was found to be influenced only by family satisfaction and work-family balance through less work-role ambiguity, while job satisfaction did not show any significant results. The presence of a significant direct effect (see Figure 5 below) also confirmed the existence of a partial mediation effect as mentioned above. 

## 5. Discussion

This study had two broad aims: (1) to test the mediating role of family satisfaction (FS), job satisfaction (JS) and work family balance (WFB) between work-family conflict (WFC) and career satisfaction (CS) and (2) between work-role ambiguity (RA) and perception of life success (LS). 

We did not find backing for H1a or H2a that tested the mediational role of FS between WFC and CS and between WFC and LS, respectively. Although WFC had a negative relationship with FS, there was no mediational relationship between FS and CS or LS. This indicates that career satisfaction and degree of life success for academic faculty may be more influenced, at this stage of their career, by satisfaction in their job and the balance they can accomplish in juggling both family and work responsibilities. This is logical as our sample consists of professionals for whom work is an integral part of their identity. Nearly 30% of our respondents acknowledged themselves as single, and many had been working only for the past 10–11 years suggesting that they are still in the phase of establishing their identity in their profession. Both these factors may have influenced the deficiency of adequate support for the role of family satisfaction in career satisfaction or perception of life success. 

As predicted in H1b and H1c respectively, job satisfaction (JS) and work-family balance (WFB) mediated between work-family conflict (WFC) and career satisfaction (CS). Similarly, H2b and H2c respectively predicted that JS and WFB mediated between WFC and perception of life success (LS). Support was found for all these four hypotheses, suggesting a reduction in WFC through increase in JS and WFB can lead to better CS and LS for faculty. It is imperative to find avenues to increase JS and feelings of competently balancing one’s work and family for faculty members. Faculty positions entail higher education and sustained job commitment over several years in order to climb the ladder of professional success. Conflict emanating from worries and impediments to sustaining family and work responsibilities may affect satisfaction in one’s job as the individual is not able to dedicate mandatory time and energy, which in turn can affect career satisfaction. Delays in publishing or presenting research and inability to commit to other professional duties in a timely fashion may lead to less job satisfaction [62]. It is vital for educational institutions to provide a robust mentoring system for faculty that can guide them, especially younger/newer faculty, in having a clearer understanding of work role demands without having to figure this out through trial and error, leading to higher job satisfaction. Such augmented job satisfaction can mitigate the possible negative influence of WFC and, instead, increase CS and LS. Similar arguments about job satisfaction reducing role stressors and increasing positive outcomes have been recorded previously [63]. Positive connection between mentoring and junior faculty job satisfaction has been documented in the west [64] but it is time that such studies commenced in non-western cultures such as India. Possibly, organizing teaching or scholarly workshops can greatly assist younger faculty members to be more in touch with the realities of teaching and conducting research from the start, instead of stepping into a faculty profession with impractical ambitions and expectations. Such constructive fallouts at work can aid faculty to better balance their work role along with their family role and curtail work conflicts with family. This can further assist in bringing about a feeling of better work-family balance leading to a healthier satisfaction in one’s career and sense of life success. 

H3a and H4a respectively tested the mediational role of family satisfaction (FS) in work-role ambiguity (WRA) and careers satisfaction, (CS) and WRA and life success (LS), respectively. While FS did not mediate between WRA and CS (H3a), support existed for H4a suggesting that FS can play a role in higher LS through less WRA. This further confirms H1a, where FS did not seem to have a relation with CS, indicating that FS by itself does not predict CS. This also alludes to the possibility that CS is primarily influenced by work-factors and FS itself does not contribute profoundly to CS. However, as H4a indicates, perception of LS can be enriched through better FS by reducing WRA. The role of social support in viewing life more positively has been documented in the literature [65]. Although family experiences of faculty are not uniform, we note the prominence of family members being perceptive regarding the responsibilities associated with a faculty position which are dissimilar to a traditional 8-5 job. Having such empathy with academic life, especially in the lives of younger faculty, can support them in becoming less overwhelmed by work role stressors including role ambiguity, thus contributing to a feeling of improved life success. 

As predicted in H3b and H3c respectively, job satisfaction (JS) and work-family balance (WFB) mediated between work-role ambiguity (WRA) and academic faculty’s career satisfaction (CS). Similarly, H4b and H4c respectively predicted that JS and WFB mediated between WRA and academic’s faculty perception of life success (LS). Job satisfaction significantly mediated between WRA and CS (H3b), but not between WRA and LS (H4b). This alludes to the substantial role job satisfaction unquestionably has in one’s career satisfaction. Having uncertainty about one’s professional duties or expectations or even professional standing can lead to less job satisfaction which can exacerbate the potential to report low career satisfaction. India scores high in power distance which specifies that there exist formal rules of conduct when dealing with one’s immediate supervisor(s) that can augment role ambiguity, as the supervisor is often not approached in order to get clarity on work-related matters. Awareness of such facts is important in finding ways to alleviate these issues so that faculty experience higher job satisfaction which can lead the way to better career satisfaction. 

Likewise, H3c and H4c were supported emphasizing that it is vital to improve a sense of balance between one’s family and work domains as this can diminish the negative experiences of WRA, leading to higher career satisfaction and a feeling of life success. Academic faculty in our sample were relatively new to their profession (average years of service was 10–11 years) indicating an early-to-mid career stage in their professional lives. Many are undoubtedly still learning the ropes of being effective and efficacious academicians. Vagueness in one’s work can have far reaching costs, including reduced creativity [66], frustration, disappointments, and mental stress including burnout [67] which can spillover to one’s family life. This may be accurate even for those respondents who identified themselves as single, as ‘family’ in India often includes extended family with the resultant obligations. Stress from work can influence such personal life obligations, ensuing in a lack of equilibrium between one’s family and work. Regrettably, this has the potential to diminish satisfaction in one’s career and in seeing one’s life as successful. Having improved sense of control in one’s work life (i.e., less role ambiguity) has been related to better work-family balance [68]. Overall, we reiterate the arguments used in our earlier discussion (of H1c and H2c) to corroborate the importance of increasing WFB among faculty and propose that experiencing enhanced WFB can contribute to faculty experiencing less WRA effect and, alternatively, feeling more LS. 

### 5.1. Theoretical and Practical Implications

This research puts forward several contributions to theory. Spillover theory has frequently been used to elucidate traditional work and family conflict or work-family balance dynamics. The current study used the framework to attempt also to enlighten how work-family conflict can have a deleterious relationship on one’s family and also on the much desired equilibrium between the two. Further, we also resorted to the use of spillover theory to elucidate how work-role ambiguity can potentially impact family satisfaction and how this in turn can negatively influence one’s career satisfaction or even one’s perception of overall success in one’s life. 

PE fit theory, not often seen in the work-family literature unlike spillover/role theories, etc., was utilized to explain several hypotheses in our research to further our comprehension of how job satisfaction or work-family balance may affect one’s career satisfaction or life success. Such analyses have furthered the utility and application of PE fit theory into realms that are understudied but warrant much consideration. Finally, we also applied the domain specific importance measure as well as subjective well-being concept to expound also on the prominence (or lack of it) of career satisfaction and sense of life success. Work-family dynamics are complex and it is our modest estimation that a single theory or concept cannot illuminate all the subtleties in these two significant domains. By utilizing the different theories and concepts, we have not only extended our understanding of work-family undercurrents but also have expanded the unique contribution of these theories/concepts in the work-family literature. 

Practically, this study further enhances our prevailing awareness of the complex world of work-family dynamics and offers tangible and hands-on knowledge catered to employees, employers and society in general. 

From an employee standpoint, we record that career satisfaction and perception of life success are multi-dimensional constructs, meaning that these are influenced by several factors. Career satisfaction is affected by various work related factors, including obscurity of duties and anticipations at work, difficulties in finding a healthy balance between work and family responsibilities and expectations, etc. Often, we presume that decent pay or reputation can bring about career satisfaction; nonetheless, factors that we habitually omit such as unpredictability associated with the job can lead to complications in work and personal lives which can affect career satisfaction in the long term. Further, although work-family conflict and work-role ambiguity and their repercussions on family and job satisfaction have been documented before, the current study also highlights that they can also correlate with a sentiment of less balance between family and job. Directly and indirectly, they can also have a bearing on the perception of life success or career satisfaction. Mindfulness of such influencing factors is the first step in taking steps to mitigate their negative influences. 

From an employer standpoint, our work puts forward suggestions to ease the negative aspects of work-family experiences for academic faculty. It is significant for organizations, particularly supervisors, to comprehend the adverse aspect of work-role ambiguity. Having a robust and competent mentoring system for newer faculty, cognizance of the fact that excessive flexibility, if unchecked, can lead to work ambiguity, and enriched clarity of expectations associated with different academic ranks, etc., can endorse a lessening in the negative spillover from work to non-work domains. This can potentially facilitate higher family and job satisfaction and improved work-family balance in addition to improving heightened career satisfaction and overall life success. 

From a society standpoint, our work brings to light the distinctive dynamics prevailing in the work-family dynamics of academic faculty in India. Family hold the foremost role in the lives of individuals in India and academic faculty are not an exception. The conclusion that poor work-family balance significantly affects career satisfaction and overall sense of life success indicates the need to endorse an awareness of assisting academic faculty in experiencing more balance between the two significant domains in their lives. Academic faculty jobs do not adhere to a strict time schedule. Work can be unpredictable as academic faculty may be anticipated to teach newer courses with all the preparatory work required, to publish, to go through the tenure process, etc. Such duties concomitant with academic faculty life are often not widely recognized or acknowledged in a society like India. This may further deteriorate the responsiveness and support that society could otherwise potentially provide to academicians. We aspire to research outcomes such as ours forming the underpinning of increasing cognizance of academic faculty life and the support that is critical to experience more positive outcomes.

### 5.2. Limitations and Future Directions

This project tested an innovative model, not yet explored in the work-family literature. The ‘exploratory research’ nature of the model limited us in finding previous, similar studies to base our arguments on or to compare our results to. We did not assess the type of work-family conflict that might affect the satisfaction and work-family balance of participants. Possibly, we might have found that distinct types of work-family conflict (for example, time or behavior) had more influence than others. We also did not evaluate work-family interface variables bi-directionally to keep our existing model simple. Bi-directional testing would have yielded distinctive results diverse from what we found in the current study. We recommend future studies on both the above points. 

This research focused only on academic faculty in India, cautioning us to not oversimplify or generalize the results which may be professional and/or culture specific. We excluded moderators, such as supervisor support or professional development opportunities, which might influence job or career satisfaction or even perception of life success. Additionally, family support or number of children and other predictors of perception of life success (such as personality traits) were not included in the current study. These factors should be considered in subsequent studies to gain a richer understanding of work-family dynamics and how they may relate to the discernment of life success.

More studies should be made in a tradition-rich and high power-distance culture such as India before the results can be generalized as holding up in non-western cultures. Current data included participants from diverse universities in India. It is highly feasible that research intensive institutions have different standards than teaching institutions, thus producing results unique to such institutions. For example, the term ‘career satisfaction’ may be interpreted differently depending on the participant’s educational qualification, their current rank and the institution they work in (research intensive versus teaching institution). We were not able to control for such differences due to the nature of the data we used to analyze the model. It is thus urged that upcoming studies control for any potential alterations pertaining to the respondents’ institutions. 

Data were collected through the survey method and limitations inherent in such a mode of collection prevail in this study, as well as participants not paying attention to survey questions or misinterpreting survey directions, their mood at the time of survey completion, the presence of any distractors in the ambiance, etc., which can affect survey responses. Further, we have used a single item to measure ‘life success’ which may have influenced the responses received. Though researchers have used single item measures to evaluate variables, a different multi-item measure to assess ‘life successes’ may yield different results.

## 6. Conclusions

The significant role of job satisfaction and work-family balance in improving career satisfaction and perception of life satisfaction was confirmed in this project. The majority of the hypotheses were supported. It is recommended that we study the nuances of work-family dynamic issues more carefully to further our grasp of the contributing factors to satisfaction and perception of success significant to the academic faculty. It is also vital that steps are undertaken by educational institutions to alleviate work-role ambiguity and work hindering fulfillment of family duties, wherever possible. We urge researchers and policy makers, especially those involved in educational institutions, to be attentive to the fact that work-family interface issues for academic faculty have their own exclusive subtleties. Thus, generalizing results (from studies done on other professionals) can be misleading. A comprehension of the unique circumstances in the work-family dynamics of academic faculty is essential and conclusions from this project present a noteworthy contribution to this.

## Figures and Tables

**Table 1:** Descriptive statistics, zero-order correlations and reliability values.

Variables	Mean	S.D.	1	2	3	4	5	6	7	8	9	10	11	12
1. Gender	0.65	0.48	-											
2. Age	34.83	9.25	0.11 *	-										
3. Marital Status	0.70	0.46	− 0.05	0.50 **	-									
4. Type of Occupation	0.76	0.43	−0.12 *	−0.36 **	−0.22 **	-								
5. Work Experience (in months)	129.17	105.28	0.10	0.94 **	0.46 **	−0.24 **	-							
6. Work-Family Conflict	2.91	0.86	0.05	−0.15 **	−0.05	0.07	−0.11 *	**0.77**						
7. Work Role Ambiguity	2.12	0.66	0.04	−0.19 **	−0.01	0.11 *	−0.17 **	0.13 *	**0.73**					
8. Family Satisfaction	4.04	0.90	0.06	0.08	0.02	−0.09	0.11 *	−0.03	−0.35 **	**0.86**				
9. Job Satisfaction	4.03	0.84	−0.11 *	0.18 **	0.10	−0.17 **	0.14 **	−0.21 **	−0.47 **	0.26 **	**0.72**			
10. Work-Family Balance	3.66	0.95	0.05	0.02	−0.03	−0.03	0.03	−0.29 **	−0.40 **	0.45 **	0.36 **	**0.75**		
11. Career Satisfaction	3.68	0.94	−0.01	0.14 **	0.05	−0.16 **	0.11 *	−0.09	−0.48 **	0.30 **	0.62 **	0.38 **	**0.74**	
12. Life Success	3.82	0.83	−0.05	0.06	0.03	−0.009	0.08	−0.06	−0.38 **	0.58 **	0.30 **	0.40 **	0.34 **	-

Note: *N* = 344, * *p* < 0.05, ** *p* < 0.01, S.D. Standard Deviation, Gender—0 Female, 1 Male, Marital Status—0 Unmarried, 1 Married, Type of Occupation—0 Government occupation, 1 Private Occupation. Cronbach’s Alpha values are reported in the diagonal with bold font.

**Table 2:** Model Fit Summary and Measurement Models Comparison.

Measurement Model	*χ* ^2^	*df*	*p*	GFI	CFI	RMSEA	AIC
Six factor model (M1)	303.17	154	<0.001	0.918	0.940	0.053	415.17
Five factor model (M2)	559.59	159	<0.001	0.859	0.839	0.086	661.59
Four factor model (M3)	914.85	163	<0.001	0.753	0.698	0.116	1008.85
Three factor model (M4)	928.26	166	<0.001	0.750	0.694	0.116	1016.26
Two factor model (M5)	1040.44	168	<0.001	0.723	0.650	0.123	1124.44
Single factor model (M6)	1153.28	169	<0.001	0.698	0.605	0.130	1235.28

Note: *χ*^2^ chi-square, *df*, degree of freedom; GFI, goodness of fit index; CFI, comparative fit index; RMSEA, root mean square error of approximation, AIC, Akaike information criterion. M1—The six factor model assumes that work-family conflict, work role ambiguity, family satisfaction, job satisfaction, work-family balance and career satisfaction are six distinct factors. M2—The five factor model has combined the job satisfaction and family satisfaction constructs into one and the rest of the constructs are treated as a distinct. M3—The four factor model has combined job satisfaction and family satisfaction as a single construct and work-family conflict and work role ambiguity as a single construct; career satisfaction and work-family balance are treated as a distinct construct. M4—The three factor model has combined job satisfaction, family satisfaction and career satisfaction as a single construct and work-family conflict, work role ambiguity as a single construct and work-family balance as a distinct construct. M5—The two factor model has combined job satisfaction, family satisfaction and career satisfaction as a single construct and work-family conflict, work role ambiguity and work-family balance as a single construct. M6—The single factor model has combined all of the constructs as a single construct.

**Table 3:** Indirect effects for work-family conflict on career satisfaction and life success.

IV: WFC Conflict	DV: Career Satisfaction			DV: Life Success		
Mediators	Indirect effect	Lower Limit	Upper Limit	Indirect effect	Lower Limit	Upper Limit
Family Satisfaction	−0.004	−0.02	0.01	−0.02	−0.07	0.03
Job Satisfaction	−0.13	−0.20	−0.07	−0.03	−0.06	−0.003
Work-Family Balance	−0.06	−0.10	−0.02	−0.04	−0.08	−0.01

**Table 4:** Indirect effects of work role ambiguity on career satisfaction and life success.

IV: Work Role Ambiguity	DV: Career Satisfaction			DV: Life Success		
Mediators	Indirect effect	Lower Limit	Upper Limit	Indirect effect	Lower Limit	Upper Limit
Family Satisfaction	−0.03	−0.09	0.02	−0.21	−0.29	−0.14
Job Satisfaction	−0.33	−0.44	−0.23	−0.04	−0.11	0.02
Work-Family Balance	−0.06	−0.13	−0.001	−0.05	−0.11	−0.004
